# Human-modified landscapes provide key foraging areas for a threatened flying mammal: The grey-headed flying-fox

**DOI:** 10.1371/journal.pone.0259395

**Published:** 2021-11-01

**Authors:** Samantha H. Yabsley, Jessica Meade, John M. Martin, Justin A. Welbergen

**Affiliations:** 1 Hawkesbury Institute for the Environment, Western Sydney University, Penrith, NSW, Australia; 2 Institute of Science and Learning, Taronga Conservation Society Australia, Mosman, NSW, Australia; University of New England, AUSTRALIA

## Abstract

Urban expansion is a major threat to natural ecosystems but also creates novel opportunities that adaptable species can exploit. The grey-headed flying-fox (*Pteropus poliocephalus*) is a threatened, highly mobile species of bat that is increasingly found in human-dominated landscapes, leading to many management and conservation challenges. Flying-fox urbanisation is thought to be a result of diminishing natural foraging habitat or increasing urban food resources, or both. However, little is known about landscape utilisation of flying-foxes in human-modified areas, and how this may differ in natural areas. Here we examine positional data from 98 satellite-tracked *P*. *poliocephalus* for up to 5 years in urban and non-urban environments, in relation to vegetation data and published indices of foraging habitat quality. Our findings indicate that human-modified foraging landscapes sustain a large proportion of the *P*. *poliocephalus* population year-round. When individuals roosted in non-urban and minor-urban areas, they relied primarily on wet and dry sclerophyll forest, forested wetlands, and rainforest for foraging, and preferentially visited foraging habitat designated as high-quality. However, our results highlight the importance of human-modified foraging habitats throughout the species’ range, and particularly for individuals that roosted in major-urban environments. The exact plant species that exist in human-modified habitats are largely undocumented; however, where this information was available, foraging by *P*. *poliocephalus* was associated with different dominant plant species depending on whether individuals roosted in ‘urban’ or ‘non-urban’ areas. Overall, our results demonstrate clear differences in urban- and non-urban landscape utilisation by foraging *P*. *poliocephalus*. However, further research is needed to understand the exact foraging resources used, particularly in human-modified habitats, and hence what attracts flying-foxes to urban areas. Such information could be used to modify the urban foraging landscape, to assist long-term habitat management programs aimed at minimising human-wildlife conflict and maximising resource availability within and outside of urban environments.

## Introduction

Urbanisation and urban expansion are major threats to ecosystems and the services they provide [1–3], due to habitat loss [4,5] and fragmentation [1], loss of biodiversity [4,6], and species extinction [7,8]. While urban growth poses ongoing threats to natural ecosystems, it can also provide new habitats, such as parks and gardens, that provide opportunities for exploitation by adaptable species [4,9]. Wildlife urbanisation can be driven by a range of ‘push’ and ‘pull’ factors. Loss of natural habitat and the resulting limitation of resources can ‘push’ animals to search for alternative resting and foraging habitat in urban environments [see 10]. Alternatively, adaptable species can be attracted to urban landscapes by novel food sources [11], reduced predation pressure [12], and lowered inter-species competition for resources [13]. However, while the presence of wildlife in urban areas can be perceived as beneficial to human physical and psychological well-being [14–16], the growing urban human-wildlife interface can result in increased human-wildlife conflict and so poses wildlife management challenges. Understanding what supports the persistence of wildlife in human-modified landscapes is thus fundamental to developing effective management responses.

In recent years, flying-foxes (*Pteropus* spp.) have become increasingly common in urban areas in Australia [17–21]. Traditionally roosts were occupied seasonally, likely reflecting the availability of floral resources in the surrounding landscape [22]. However, many urban roosts are now occupied year-round [18]. Flying-fox urbanisation has been hypothesised to be a result of loss of native habitat and urban expansion [23,24], and increases in the availability [17,18,23] and temporal stability of urban food resources [17,18,23,25,26] due to planting of native and exotic trees [27,28].

Flying-foxes are of critical ecological importance [29] delivering long distance pollination and seed dispersal services that maintain the health and diversity of native habitats, connect forest fragments, and aid in forest regeneration [30,31]. Despite this, their presence in urban landscapes has become a prominent management issue [32–34]. Urban human versus flying-fox conflicts arise from concerns around transfer of zoonotic diseases [35], noise, smell, and faeces [34], depredation of fruit crops including backyard fruit trees [36–38], and the defoliation of roosting trees [39]. Current management strategies include the removal of roosting trees, dispersal of flying-foxes from their roosts by means of smoke and noise [33,40], and even culling [41,42]. However, these methods have had limited success and often inadvertently exacerbate the human-wildlife conflict they aim to resolve. For example, forced dispersals of roosting flying-foxes can lead to the formation of splinter colonies and so proliferate the human-wildlife conflict throughout human communities [33] and the stress induced by roost dispersal may increase the prevalence of zoonotic disease and the risk of spillover events [43]. Culling by direct shooting also raises particular animal welfare concerns, as shooting often results in injuries that cause long-term suffering, and can leave dependent young of shot mothers to die of starvation [e.g. 41]. Besides raising animal welfare issues, culling of flying-foxes also fails to mitigate drivers of human-versus flying-fox conflict including fruit crop predation [e.g. 44]. Understanding what foraging resources support flying-foxes in human-modified landscapes throughout time and space will thus help managers make informed decisions regarding humane conflict mitigation and conservation of these ecologically important species.

The grey-headed flying-fox (*Pteropus poliocephalus*) is one of four mainland flying-fox species native to Australia, and is listed as Vulnerable under the IUCN Red List [45] and Australia’s federal legislation [46]. Like other flying-foxes in Australia and elsewhere, this species has become increasingly urbanised [17,18,28], and this exposes the species to human-wildlife conflict [33,40], along with other anthropogenic threats such as electrocution on power lines and entanglement in fruit tree netting [47–50]. Recent research has indicated that urban roosting *P*. *poliocephalus* exhibit higher roost fidelity and have shorter foraging distances than where they roost in non-urban habitat, supporting the hypothesis that urban areas provide more favourable foraging conditions than non-urban areas [51]. However, at present little is known about how *P*. *poliocephalus* use the urban landscape for foraging, including how they use foraging sites across time and space, and the composition of their diet in urban areas. As such, we do not know what supports flying-fox urbanisation, which poses serious impediments for the management and conservation of this threatened species [52]. To investigate whether *P*. *poliocephalus* foraging landscape utilisation differs between urban and non-urban landscapes, we used satellite tracking data for 98 individuals tracked throughout New South Wales (NSW), Australia, for up to five years. In particular, we examined the foraging preferences of urban and non-urban roosting *P*. *poliocephalus* according to vegetation type and the likely tree species that foraging individuals visited, and according to a published index of flying-fox foraging habitat quality. We discuss our findings in the context of the management of flying-fox urbanisation.

## Materials and methods

### Capture and deployment of transmitters

*Pteropus poliocephalus* were captured at the Royal Botanic Garden roost (33.8642°S, 151.2166°E), in Sydney, New South Wales (NSW), Australia from 9th-18th May 2012. Capture was conducted pre-dawn as *P*. *poliocephalus* returned to the roost, using mist nets (12 m x 4.8 m; mesh size 20 mm) suspended by two 15 m aluminum poles. Caught individuals were restrained and untangled immediately after capture. Captured individuals were assessed for sex, age, and body condition, and then placed into individual pillowcases suspended from horizontal poles, for processing that morning. Upon processing, detailed body measurements were taken, and 49 male and 50 female *P*. *poliocephalus* with no injury/illness and weighing ≥ 650 g were anesthetized using the inhalation agent Isoflurane [53] and then fitted with transmitters. The transmitter package consisted of a collar-mounted solar satellite transmitter, attached to a neoprene-lined leather collar, and fastened by a rivet. Microwave Telemetry 9.5 g transmitters were deployed on females and GeoTrak 12 g transmitters were deployed on males [51,54]. The total combined mass of the collar and transmitter was < 15 g which corresponded to < 3% of the body mass of the lightest individual in the sample (n = 98). Individuals were released at the capture site upon recovery, by midday, after being offered fruit juice for energy and hydration.

Transmitter duty cycles varied; the ‘on’ period was always set to 10 h, but the ‘off’ period was set to a range of values from 50 h to 254 h off, to maximize opportunities for solar recharge. During the ‘on’ periods, locational data was transmitted to orbiting NOAA satellites and sequentially received via ARGOS.

Fieldwork was approved and conducted under the Office of Environment and Heritage Animal Ethics Committee permit 110620/05 and Scientific License 100268.

### Data handling and analysis

This study analysed *P*. *poliocephalus* satellite tracking data collected between 9th May 2012–27th April 2017 in NSW. Data were subsetted such that of 100,463 data points, all 51,585 high quality ARGOS location data classes 2 and 3 were initially retained. Positional fixes of these classes are estimated to be accurate to within 250 m and 500 m of the true location, respectively [55]. To investigate foraging locations, following [51], we selected all positional fixes collected during the 10 h ‘on’ periods for which both daytime and night-time location data were available. The daytime fix allowed the roosting colony to be identified, and the night-time fix furthest from the roost site was selected as the assumed foraging location. This resulted in 5,118 paired roosting and foraging locations. Next, we excluded all paired locations (n = 52) where their distance was greater than 50 km, as 99% of foraging takes place within 50 km from a roost [54,56,57] so that greater distances likely represent movements between roosts. Recent research suggests that *P*. *poliocephalus* individuals travel directly to a foraging site early in the night and then undertake smaller movements between foraging sites before returning to the roost [28]. Thus, while we cannot be certain that these locations are ‘foraging locations’ it is likely that the location furthest from the roost site in a night is in an area that an individual was foraging. Finally, we subsetted the data to those animals foraging in NSW to allow for comparison with available data layers (below), resulting in 4,198 paired roosting and foraging locations for 98 of the 99 tracked individuals in this study area.

#### Data layers

Land-use categories were extracted for each of the foraging locations in NSW (n = 4,198). For this we used a shapefile of Urban Centre and Locality data obtained from the Australian Bureau of Statistics [58], to classify NSW into three land-use categories. Land was defined as ‘of urban character’ based on dwelling density and population density [59]. ‘Major-urban’ areas were defined as urban centers with a population of > 100,000 [59]. ‘Other-urban’ areas were urban centers with a population of between 1,000 and 99,999 [59]. All other areas were defined as ‘non-urban’. For ease of interpretation, we refer to the ‘other-urban’ land-use category as ‘minor-urban’ throughout.

Vegetation types were extracted for each of the foraging locations in NSW (n = 4,198). For this we used the Vegetation Formations and Classes of NSW (version 3.03–200 m Raster) to classify vegetation type in NSW to 16 core classes [60]. The raster was created and published in 2012 and is thus concurrent with our tracking data. In the Vegetation Formations and Classes of NSW, ‘cleared land’ is defined as land that is not structurally intact native vegetation [61]. Thus, cleared land comprises human-modified land including agriculture, parks, gardens, and tree-lined streets. Cleared land may also include small remnant patches of native vegetation up to 2 hectares. For clarity, we refer to ‘cleared land’ as ‘human-modified land’ henceforth.

Flying-fox foraging habitat quality ranks from Eby and Law [62] were available for 3,757 of the n = 4,198 foraging locations in NSW. These habitat quality ranks are based on a complex algorithm incorporating the spatial availability of known *P*. *poliocephalus* blossom food plant species and indices of productivity and nectar flow, as well as species richness scores of fruit food plant species [62]. Here, habitat quality was ranked from 1 (high quality) to 4 (poor quality), and areas were ranked as 0 if neither the dominant nor subdominant species were known *P*. *poliocephalus* food plant species. We extracted likely food plant species from vegetation shapefiles from Eby and Law [62]. These vegetation shapefiles only contained food plant species in the blossom diet of *P*. *poliocephalus*, as insufficient data were available on the productivity and reliability of food plant species in the fruit diet [62]. Eby and Law’s diet plant list comprised 59 species in the blossom diet including species from the Myrtaceae, Proteaceae, Arecaceae, Fabaceae, and Pittosporacea families. Only dominant and sub-dominant species [63] were considered resulting in a list of 55 species (see Table 4.1 in [62]). Habitat quality rank data were split into bi-months to account for seasonal variations in flowering phenology of the food plant species [62]; December-January, February-March, April-May, June-July, August-September, October-November. Where more than one dominant or sub-dominant food plant species was available in the bi-month that a foraging fix was recorded, the species that flowered most often and that was most abundant was selected as the most likely food plant species [62]. We used a shapefile of *P*. *poliocephalus’* range [40] and of NSW [64] to clip all data layers.

#### Analysis

Preliminary analyses revealed that the proportion of foraging fixes in each vegetation type did not differ significantly between study years (the cut-off between years was May 9th as this is when catching began) (Friedman χ^2^ = 6.79, df = 4, p = 0.147), bi-month (Friedman χ^2^ = 2.19, df = 5, p = 0.823), or between sexes (Friedman χ^2^ = 0.818, df = 1, p = 0.366). Similarly, the proportion of foraging fixes in each habitat quality rank did not differ significantly between years (Friedman χ^2^ = 2.40, df = 4, p = 0.663), between bi-months (Friedman χ^2^ = 5.00, df = 5, p = 0.416), or between sexes (Friedman χ^2^ = 1.80, df = 1, p = 0.180). Therefore, the data were analysed as a whole.

To examine whether *P*. *poliocephalus* exhibited preferences for certain foraging habitats we compared the proportion of foraging fixes in each vegetation type to the proportion that would be expected based on the area of each vegetation type available in *P*. *poliocephalus’* range inside NSW, using a chi-squared test for given probabilities. To examine whether flying-foxes have a preference for high quality foraging habitat [i.e. ranks 1 and 2; 62], this process was repeated for the areas in which a habitat quality rank was available (see Fig 6.7 in [62]).

Finally, non-urban roosting locations varied in their distance from the nearest urban polygon (S1 Fig). To test for an effect of distance to urban polygons on the relative frequencies of different vegetation types visited by foraging animals, we performed multinomial logistic regression using the ‘multinom’ function from the R package ‘nnet’ [65].

All analyses were performed in the R environment for statistical computing [66].

## Results

**Fig 1:**
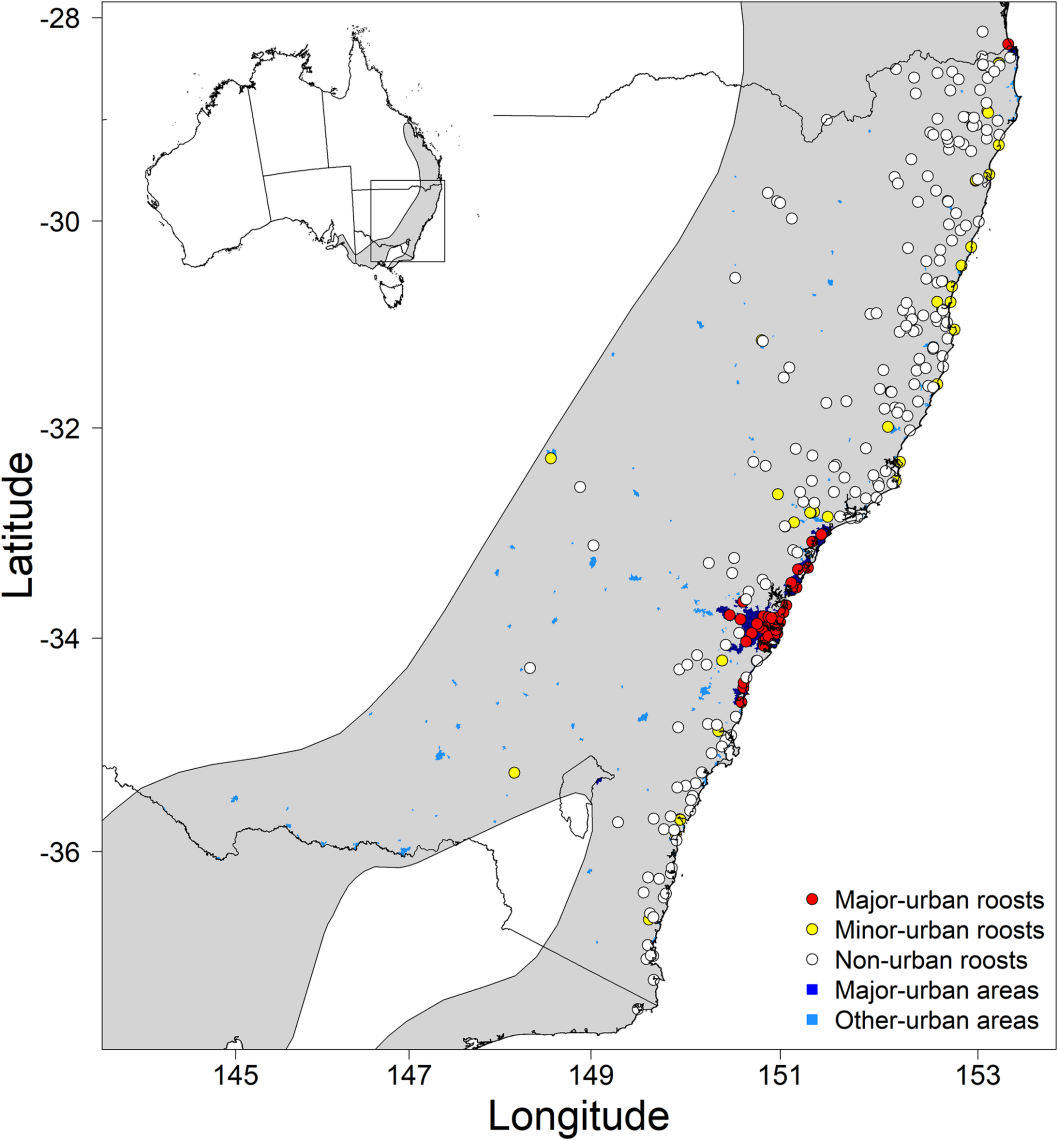
Map of *P*. *poliocephalus* roost sites. Red points indicate the location of roosts in major-urban areas, yellow dots indicate roosts in minor-urban areas, and white dots indicate roosts in non-urban areas. Major-urban areas are indicated by dark blue shading, minor-urban areas are indicated by mid-blue shading. Lines indicate State boundaries, and *P*. *poliocephalus’* range. Shaded grey area shows *P*. *poliocephalus’* range in Australia. Inset, map of Australia with box indicating area used in this study.

Overall, 4,198 foraging fixes were identified from 98 *P*. *poliocephalus* individuals that roosted at 263 unique roosts within NSW over a period of up to five years. Of the 263 unique roosts, 31 (11.8%) occurred in NSW’s major-urban areas, 37 (14.1%) in minor-urban areas, and 195 (74.1%) were located in non-urban areas (Fig 1). Of the 98 tracked individuals, 46 roosted in all three land-use categories during their tracking periods (see S1 Table for further details).

### Vegetation type preferences

The spatial distribution of foraging fixes (n = 4,198) was significantly different to the proportion that would be expected based on the areal extent of available vegetation types overall (χ^2^ = 1401, *df* = 15, p < 0.001), indicating that individuals preferentially visited certain vegetation types over others (S2 Fig).

**Fig 2:**
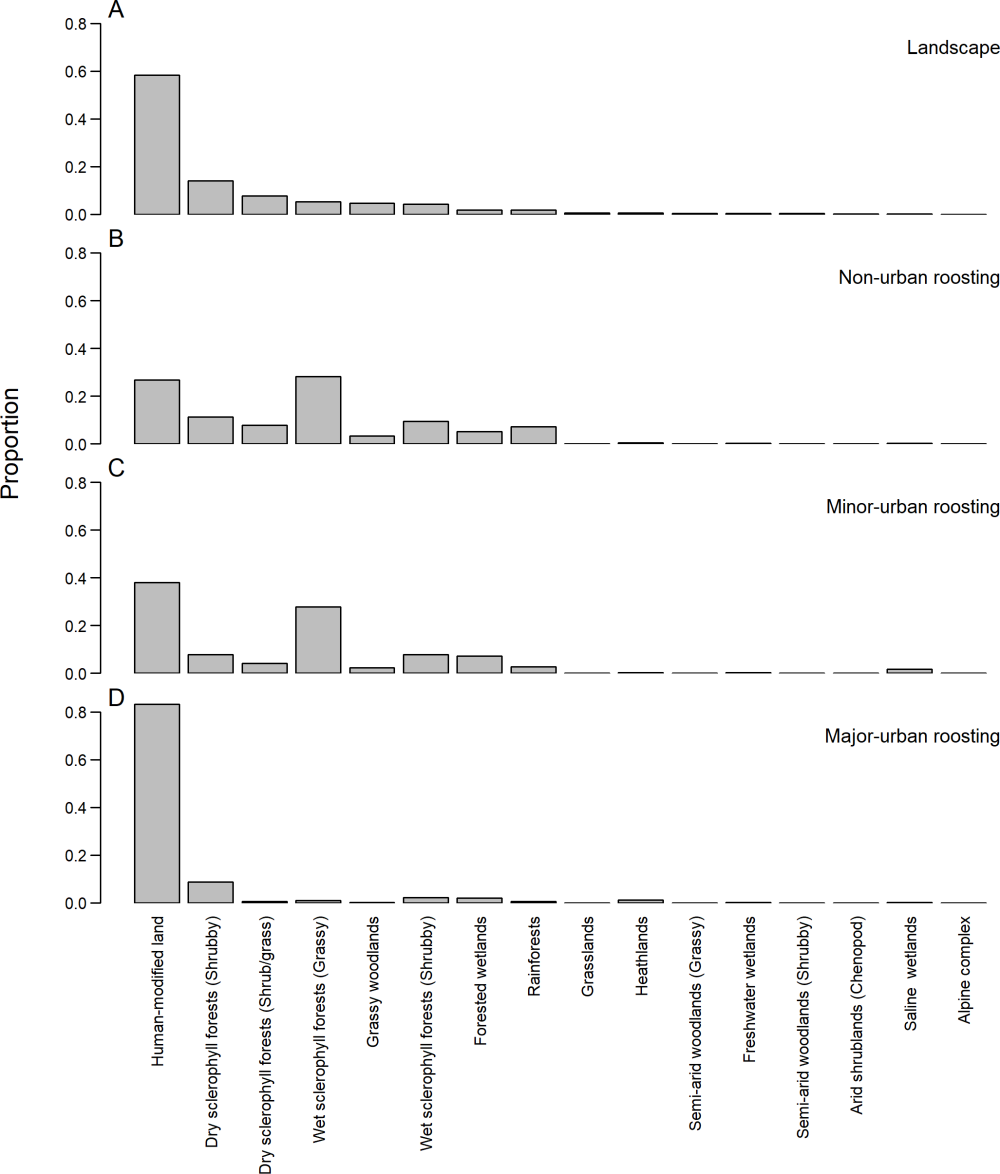
Vegetation class preferences. The proportion of (A) each vegetation class in the study area, and (B) of positional fixes (n = 1,236) recorded from *P*. *poliocephalus* roosting in non-urban colonies in each vegetation class, (C) of positional fixes (n = 974) recorded from *P*. *poliocephalus* roosting in minor-urban colonies in each vegetation class, (D) of positional fixes (n = 1,988) recorded from *P*. *poliocephalus* roosting in major-urban colonies in each vegetation class.

The majority of foraging fixes occurred in human-modified land (56%), which was similar to the proportion of human-modified land within *P*. *poliocephalus*’ range in NSW (58%). Tracked individuals exhibited a preference for wet sclerophyll forests (grassy subformation), since this made up 15% of all foraging fixes despite only covering 5% of the study area. However, when foraging locations were divided up based on the land-use category of where the animals were roosting [major-urban: n = 1,988 (47.6%); minor-urban: n = 974 (23.2%); and non-urban: n = 1,236 (29.4%)], the results revealed stark differences between the land-use categories of roosting locations. When roosting in non-urban and minor-urban areas, individuals foraged less in human-modified land than would be expected based on areal availability (26% and 38% of foraging fixes, respectively, vs 58% of area available in NSW; Fig 2A–2C) and showed a preference for wet sclerophyll forests (grassy subformation) (both 28% of foraging fixes vs 5% of area available in NSW; Fig 2A–2C). In contrast, when roosting in major-urban areas individuals foraged overwhelmingly in human-modified areas (83% of foraging fixes vs 58% of area available in NSW; Fig 2D).

**Fig 3:**
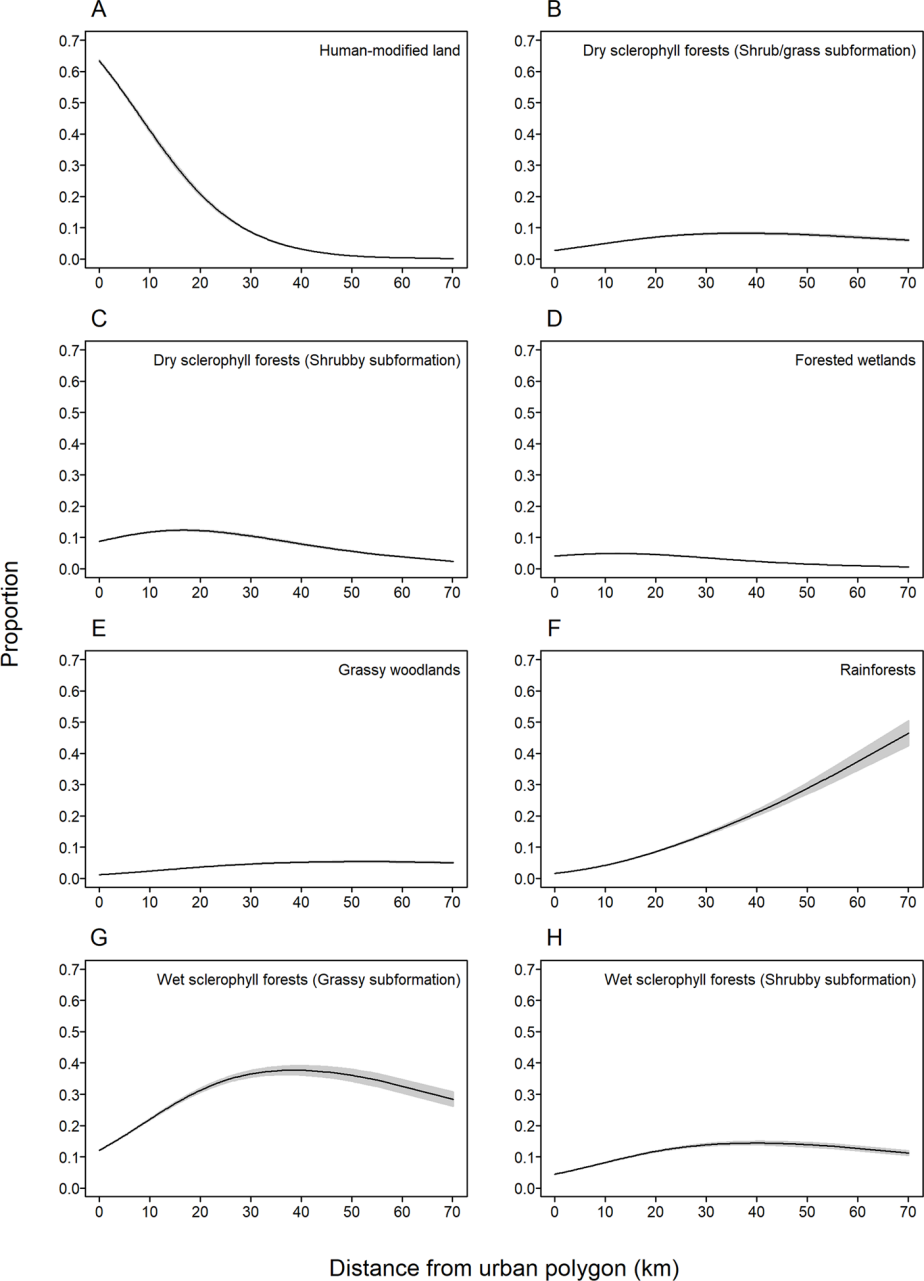
Predicted changes in vegetation class preferences with distance from urban areas. The predicted change in proportion of vegetation types visited by foraging *P*. *poliocephalus* roosting at non-urban colonies as the distance from the roost site to the nearest urban polygon increases. Predictions are taken from a multinomial logistic regression model. Grey polygons indicate 95% confidence intervals. Vegetation types that made up <1% of foraging fixes were excluded from the graph.

We found an effect of distance to the nearest urban polygon on the relative frequency of the vegetation types visited by foraging individuals (AIC of 11988.1 *vs* AIC of 12538.2 for null model, evidence ratio >1000). As the distance to urban polygons increased, the proportion of human-modified land visited decreased (Fig 3A), and the proportion of rainforest area visited increased (Fig 3F). The proportion of several vegetation types visited remained consistently low, irrespective of distance to the nearest urban polygon (Fig 3D and 3E. The proportion of visits to the four types of sclerophyll forests peaked when roost sites were 15–40 km from the nearest urban polygon (Fig 3B, 3C, 3G and 3H) (see S2 Table for more details).

### Habitat quality rank preferences

**Fig 4:**
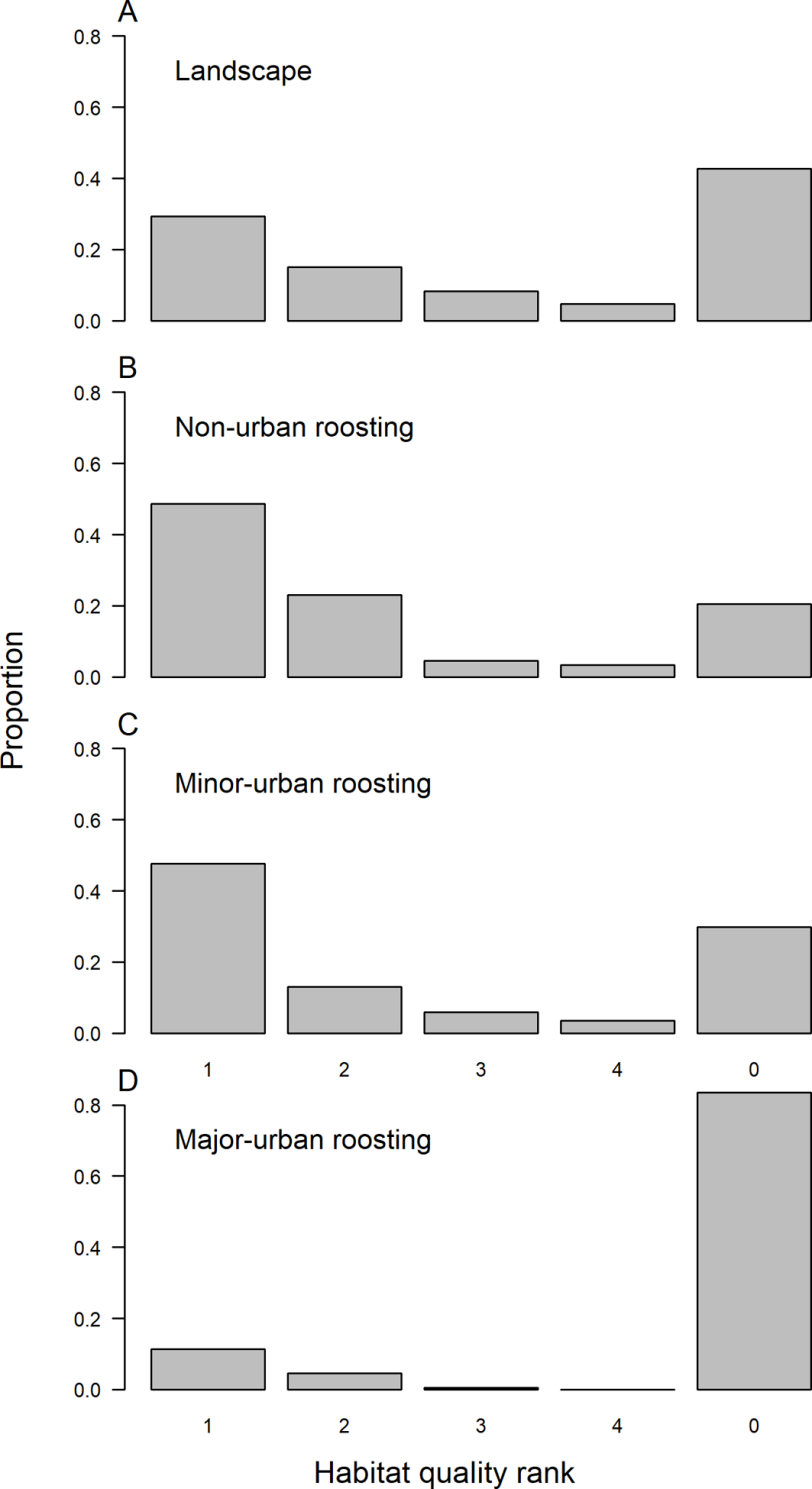
Habitat quality preferences. The proportion of (A) each habitat quality rank in the sampled area, (B) of positional fixes (n = 1031) recorded from *P*. *poliocephalus* roosting in non-urban colonies in each habitat quality rank, (C) of positional fixes (n = 770) recorded from *P*. *poliocephalus* roosting in minor-urban colonies in each habitat quality rank, and (D) of positional fixes (n = 1956) recorded from *P*. *poliocephalus* roosting in major-urban colonies in each habitat quality rank. Habitat quality was ranked from 1–4: where 1 is good quality foraging habitat, rank 4 is poor quality foraging habitat, and rank 0 is habitat where the recorded dominant and subdominant plant species are not part of the *P*. *poliocephalus* diet.

The spatial distribution of foraging fixes for which habitat quality ranks were available (n = 3,757) was significantly different to the proportion that would be expected based on the areal extent of these habitats (χ^2^ = 381.1, *df* = 4, p < 0.001; S3 Fig); the main difference being that a greater than expected proportion of foraging fixes occurred in areas where the recorded dominant and subdominant plant species were not part of the *P*. *poliocephalus* diet. However, when foraging locations were divided up based on the land-use category of where the animals were roosting, results revealed stark differences between land-use categories of roosting locations: individuals roosting in non-urban and minor-urban areas visited a greater proportion of high-quality habitat (rank 1) than was available in the landscape (Fig 4A–4C; 49% & 48% respectively vs 29% for the sampled area), whereas the vast majority (83%) of foraging fixes of major-urban roosting individuals were in areas where the recorded dominant and subdominant plant species are not part of the *P*. *poliocephalus* diet (rank 0) (Fig 4D).

### Food plant species

We used Eby and Law’s [62] habitat layers to extract likely native, dominant and sub-dominant *P*. *poliocephalus* food plant species for each foraging fix that was in the sampled area (n = 3,757). Of these, 71% were in areas where the dominant and subdominant plant species recorded flowering in the bi-month that the forage fix was observed, were not part of the *P*. *poliocephalus* diet (major-urban roosting: n = 1,811; minor-urban roosting: n = 376; non-urban roosting: n = 471). Thus, Eby and Law’s [62] data are limited in urban, particularly major-urban areas.

**Fig 5:**
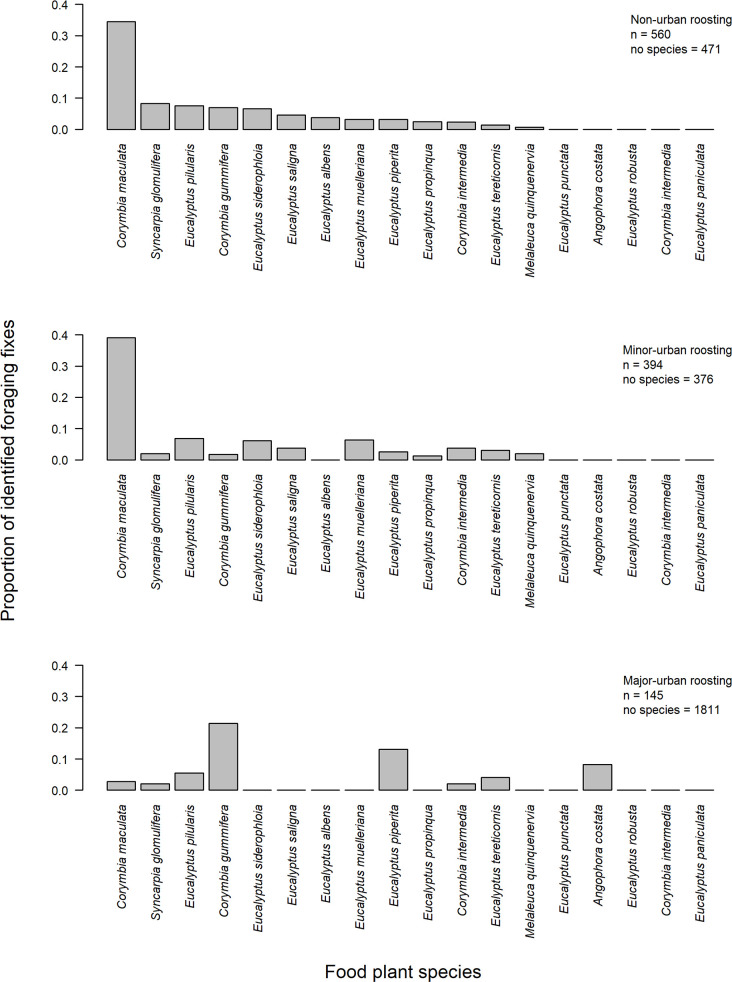
Likely food plant species. The identified likely food plant species overall when *P*. *poliocephalus* roosted in non-urban, minor-urban and major-urban areas. A maximum of 18 different food plant species were included in each graph. n = the number of foraging fixes used to calculate proportions. No species indicate additional fixes for the particular bi-month that fell in the sampled area, but for which the recorded dominant and subdominant plant species were not part of the *P*. *poliocephalus* diet.

Examining the food plant species in more detail, tracked individuals appeared to exploit similar plant species when roosting in non-urban and minor-urban habitats (Figs 5 and S4A and S4B) but different plant species when roosting in major-urban areas (Figs 5 and S4A–S4C). Individuals roosting in major-urban areas foraged on a greater proportion of *Corymbia gummifera*, *Eucalyptus piperita*, and considerably less on *C*. *maculata* overall, than when roosting in non-urban and minor-urban habitats (Figs 5 and S4A–S4C). However, note that overall, sample sizes were relatively small as only 145 (7%), 394 (51%), and 560 (54%) foraging fixes from major-urban, minor-urban, and non-urban roosting flying-foxes could be assigned a likely food plant species, respectively (Figs 5 and S4A–S4C).

The top three likely food plant species assigned to foraging fixes where individuals roosted in non-urban habitat during each bi-month included *E*. *pilularis* (n = 24, 0.273), *E*. *piperita* (n = 15; 0.170), and *C*. *gummifera* (n = 8; 0.091) in December-January; *C*. *gummifera* (n = 31, 0.223), *E*. *saligna* (n = 20, 0.144) and *E*. *muelleriana* (n = 17, 0.122) in February-March, *C*. *maculata* (n = 57, 0.838); *E*. *grandis* (n = 6, 0.088) and *E*. *robusta* (n = 3, 0.044), in April-May; *C*. *maculata* (n = 63, 0.851), *E*. *albens* (n = 5, 0.068) and *E*. *pilularis* (n = 4, 0.054) in June-July; *C*. *maculata* (n = 67, 0.583), *E*. *siderophloia* (n = 19, 0.165) and *E*. *albens* (n = 16, 0.139) in August-September; and *Syncarpia glomulifera* (n = 40, 0.526), *E*. *siderophloia* (n = 15, 0.197) and *E*. *planchoniana* (n = 6, 0.079) in October-November (S4A Fig).

The top three likely food plant species assigned to foraging fixes where individuals roosted in minor-urban habitat during each bi-month included *E*. *paniculata* (n = 20, 0.189), *E*. *pilularis* (n = 16, 0.151), and *E*. *muelleriana* (n = 11, 0.104) in December-January; *E*. *muelleriana* (n = 14, 0.222), *E*. *paniculata* (n = 10, 0.159), and *E*. *pilularis* (n = 8, 0.127) in February-March; *C*. *maculata* (n = 46, 0.807), *Melaleuca quinquenervia* (n = 4, 0.070), and *E*. *pilularis* (n = 3, 0.053) in April-May; *C*. *maculata* (n = 87, 0.926), *E*. *tereticornis* (n = 4, 0.043), and *M*. *quinquenervia* (n = 3, 0.032) in June-July; *C*. *maculata* (n = 21, 0.538), *E*. *siderophloia* (n = 12, 0.308), and *S*. *glomulifera* (n = 3, 0.077) in August-September; and *E*. *paniculata* (n = 10, 0.286), *E*. *siderophloia* (n = 8, 0.229), and *E*. *tereticornis* (n = 6, 0.171) in October-November (S4B Fig).

The top three likely food plant species where individuals roosted in major urban habitat in each bi-month included *E*. *punctata* (n = 13, 0.464), *E*. *piperita* (n = 6, 0.214) and *E*. *pilularis* (n = 5, 0.179) in December-January; *C*. *gummifera* (n = 30, 0.380), *E*. *punctata* (n = 30, 0.380), and *E*. *piperita* (n = 13, 0.165) in February-March; *E*. *robusta* (n = 5, 0.556) and *C*. *maculata* (n = 4, 0.444) in April-May, *E*. *robusta* (n = 5, 1) in June-July; *E*. *paniculata* (n = 3, 0.75) and *E*. *tereticornis* (n = 1, 0.25) in August-September; and *Angophora costata* (n = 12, 0.6), *E*. *tereticornis* (n = 5, 0.25), and *Syncarpia glomulifera* (n = 3, 0.15) in October-November (S4C Fig).

## Discussion

We used a large satellite tracking dataset of 98 individual *P*. *poliocephalus* over up to 5 years to examine foraging landscape utilisation for animals roosting across 263 roosts in urban and non-urban areas in NSW. The findings demonstrate clear differences in urban- and non-urban landscape utilisation in foraging *P*. *poliocephalus* individuals, and indicate that human-modified landscapes, including agriculture, parks, gardens, tree-lined streetscapes, and remnant patches of native vegetation, provide important foraging resources for the species, particularly in major urban areas.

Individuals roosting in non-urban and minor-urban areas visited similar vegetation types, comprising mainly of wet and dry sclerophyll forests, forested wetlands, and rainforest (Fig 2), and preferred high-quality foraging habitat (Fig 4), in line with known natural foraging preferences of the species [62,67,68]. However, while non-urban and minor-urban roosting flying-foxes foraged less in human-modified land than would be expected based on areal availability, human-modified land still encompassed 26% and 38% of their foraging fixes, respectively (Fig 2), albeit the relative contribution of human-modified lands for foraging individuals diminished with distance of their roosts to the nearest urban polygon (Fig 3). Thus, while the landscape utilisation of non-urban and minor-urban roosting individuals aligned well with the known natural foraging ecology of the species, our findings also highlight the importance of human-modified foraging areas for individuals roosting outside major-urban areas.

Human-modified foraging landscapes were particularly important for flying-foxes roosting in major-urban areas, with an overwhelming majority of their foraging fixes occurring in human-modified areas. A large proportion of these foraging fixes occurred in habitat rank 0; habitat where the vegetation comprised neither dominant nor subdominant listed *P*. *poliocephalus* food plant species. For the list of known food plant species used in this study we used the data provided by [62] that includes only native species that *P*. *poliocephalus* have been recorded feeding on from field observations and/or identified through faecal analysis. However, non-dominant species available in rank 0 habitats may include species on the *P*. *poliocephalus* diet list, and/or include non-endemic or exotic food plant species of which we currently do not know the importance to the *P*. *poliocephalus* diet. In addition, in the data provided by [62], food plant species in the fruit diet of *P*. *poliocephalus* are underrepresented because i) these almost exclusively existed in rainforest which made up just 2% of the study area, and ii) there was insufficient data on the phenology of food plants in the fruit diet of *P*. *poliocephalus* [62]. Nevertheless, previous studies from Melbourne [26] and Adelaide [28] suggest that *P*. *poliocephalus* forages on the blossom and fruit of a mixture of native and non-endemic plant genera growing in streetscapes, parks, and gardens, and it has been hypothesised that historical increases in the spatiotemporal availability of these foraging resources have facilitated the expansion of the species into urban areas [17]. This is supported by recent findings that while *P*. *poliocephalus* individuals exhibit extreme mobility among roosts throughout the species’ range [54] forage over shorter distances when roosting in major-urban areas [51] implying that urban roosting flying-foxes are supported by a more stable and abundant supply of local foraging resources. Of the 263 unique roosts visited in our present study, only 11.8% were classified as major-urban (Fig 1), yet these were associated with 47.6% of all foraging fixes of which 83% were in human-modified areas (Fig 2). Therefore, these results highlight the importance of both urban roosts and associated human-modified foraging areas for supporting a large yet dynamic proportion of the threatened *P*. *poliocephalus* population.

Though limited, the available data on likely forage species in this study suggested that individuals foraged on different plant species when roosting in major-urban areas versus when roosting in minor-urban and non-urban areas (Fig 5). Our results may simply reflect the different plant resources available to this generalist species in urban centers compared to more natural habitat; however, it could also reflect a greater availability of urban nectar, pollen and/or fruit resources due to more regular and intense flowering. Australian plants are notorious for their irregular flowering, some with intervals of up to several years [69]. Interestingly, 60% of the most likely forage species assigned to flying-foxes roosting in major-urban areas during October-November was *A*. *costata*, while this species was not associated with foraging individuals that roosted outside of major-urban areas. Previous research found that *A*. *costata* only flowered within street habitats and did not flower at all in remnant and open forests, however this was a short-term study with a small sample size [70]. The authors proposed, in support with the wider literature [71], that the urban heat island effect may be responsible for these differences in urban and non-urban phenologies. Other factors associated with urban environments, including higher soil moisture and soils containing higher phosphorus and nitrogen levels as a result of urban runoff of fertilisers and stormwater [70] and increased water availability [72] could affect growth and flowering of urban trees and so increase the spatiotemporal availability of food for flying-foxes in urban areas [11,17]. However, further research is needed to understand how and why the urban foraging landscape differs from that in non-urban environments, to help explain what attracts flying-foxes to urban areas.

Our results suggest that *P*. *poliocephalus* may not be attracted to minor-urban roosts because of the availability of urban foraging resources, as foraging landscape utilisation of individuals roosting in minor-urban and natural areas was similar (Fig 2). It is possible that minor-urban roosts instead provide more protection from predators [73], including white-bellied sea eagles (*Haliaeetus leucogaster*), wedge-tailed eagles (*Aquila audax*), and powerful owls (*Ninox strenua*). Alternatively, *P*. *poliocephalus* may roost in small towns due to climate effects [74], or proximity to water [75]. It is also possible that minor-urban areas may provide some navigational benefit due to landmarks or lighting [e.g. 28]. Finally, perhaps roosting in smaller towns is merely an incidental by-product of a learnt association between urban development and increased foraging success experienced by individuals that also forage in major-urban areas. At present, however, the reasons why flying-foxes roost in smaller towns remain unclear [20] and requires further investigation.

The reliance of *P*. *poliocephalus* on human-modified areas may have important negative implications for this threatened species. Animals including flying-foxes may be attracted to the increased foraging resource availability in major-urban areas; however, such habitats may act as ‘ecological traps’ [76]. Major-urban areas present a range of challenges to *P*. *poliocephalus* including human-wildlife conflict [40,77], and other anthropogenic threats such as electrocution on power lines and entanglement in fruit tree netting in gardens [47–50]. In addition, increasing frequency and severity of extreme heat events in Australia has caused mass mortality of flying-foxes due to hyperthermia [78], and urban colonies may be more exposed to the effects of these events due to the urban heat island effect [79]. Foraging in human-modified land outside of major-urban areas also exposes the species to anthropogenic threats, including increased conflict with fruit growers [37,80] that can result in culling [41,81], and entanglement in commercial fruit netting and barbed-wire fences [47,48,50]. Flying-foxes have a low natural reproductive ability [82], which renders them particularly vulnerable to population declines from such anthropogenic threats, especially those operating at landscape-scales. Yet, there has been little scope for flying-foxes to adapt to these novel challenges, particularly as flying-fox urbanisation as a biological phenomenon has only been occurring over the last two decades or so [19], comprising approximately three flying-fox generations [83]. Future research should quantify the impacts on *P*. *poliocephalus* particularly of threats associated with human-modified landscapes, to inform both conservation management and human-wildlife conflict mitigation.

Further research is clearly needed to identify the exact foraging resources that support flying-foxes, particularly in major-urban areas. High resolution GPS tracking is a good candidate for this since these data are accurate to the scale of an individual tree. In major-urban areas this would reveal which food plant species support the large urban flying-fox populations, and elucidate whether urban populations are supported mainly by exotic trees or by native species that are able to flower or fruit more abundantly and/or for longer due to favourable urban growing conditions [72]. In addition, it could provide targets for government-subsidized exotic tree removal, to minimise foraging in conflict zones and help reduce anthropogenic risks to flying-foxes. However, while exotic tree removal is considered an effective measure for reducing local human versus flying-fox conflict [84], it would raise serious concerns for the *P*. *poliocephalus* population as a whole, as it could result in an overall reduction in the foraging resource base for this species, unless local tree removal is offset by targeted plantings of forage trees in natural areas. Clearly, sound, long-term conservation management of *P*. *poliocephalus* needs to be predicated on better knowledge of its foraging landscape utilisation, to enable more holistic, coordinated habitat management programs that focus on redirecting flying-fox foraging away from urban conflict areas whilst enhancing and restoring resource availability elsewhere for this vulnerable species.

## Supporting information

S1 Fig Distribution of non-urban roosts in relation to the distance to the nearest urban polygon. (TIF)

S2 Fig The proportion of (A) each vegetation class in the study area, and (B) of *Pteropus poliocephalus* foraging positional fixes (n = 4,233) recorded in each vegetation class. Satellite tracking data was collected between 2012–2017 and is representative of 98 individuals.
(TIF)

S3 Fig The proportion of (A) each habitat quality rank in the area sampled, and (B) of foraging positional fixes (n = 3,773) recorded in each habitat quality rank. Habitat quality was ranked from 1–4: where 1 is good quality foraging habitat, rank 4 is poor quality foraging habitat, and ‘No species’ is habitat where the recorded dominant and subdominant plant species are not part of the known *P*. *poliocephalus* diet.
(TIF)

S4 Fig A. Likely *P*. *poliocephalus* food plant species in each bi-month where individuals roosted in non-urban areas. A maximum of 10 different food plant species were included in each graph. n = the number of foraging fixes used to calculate proportions. ‘No species’ indicate additional fixes for the particular bi-month that fell in the sampled area, but for which the recorded dominant and subdominant plant species were not part of the known *P*. *poliocephalus* diet. B. Likely *P*. *poliocephalus* food plant species in each bi-month where individuals roosted in minor-urban areas. A maximum of 10 different food plant species were included in each graph. n = the number of foraging fixes used to calculate proportions. ‘No species’ indicate additional fixes for the particular bi-month that fell in the sampled area, but for which the recorded dominant and subdominant plant species were not part of the known *P*. *poliocephalus* diet. C. Likely *P*. *poliocephalus* food plant species in each bi-month where individuals roosted in major-urban areas. A maximum of 10 different food plant species were included in each graph. n = the number of foraging fixes used to calculate proportions. ‘No species’ indicate additional fixes for the particular bi-month that fell in the sampled area, but for which the recorded dominant and subdominant plant species were not part of the known *P*. *poliocephalus* diet.
(TIF)

S1 Table The numbers of visiting individuals for all combinations of roost types (N_total_ = 98 individuals, satellite tracked over up to 5 years between 2012–2017). (TIF)

S2 Table Estimated regression parameters, standard errors, z values, and p-values for the best fitting multinomial logistic regression. Cleared land is the reference category.
(TIF)
