# Supplementary figures and images for: Human-modified landscapes provide key foraging areas for a threatened flying mammal: The grey-headed flying-fox
Source: PLoS One. 2021 Nov 1;16(11):e0259395. doi: 10.1371/journal.pone.0259395 (PMC8559981; doi:10.1371/journal.pone.0259395)

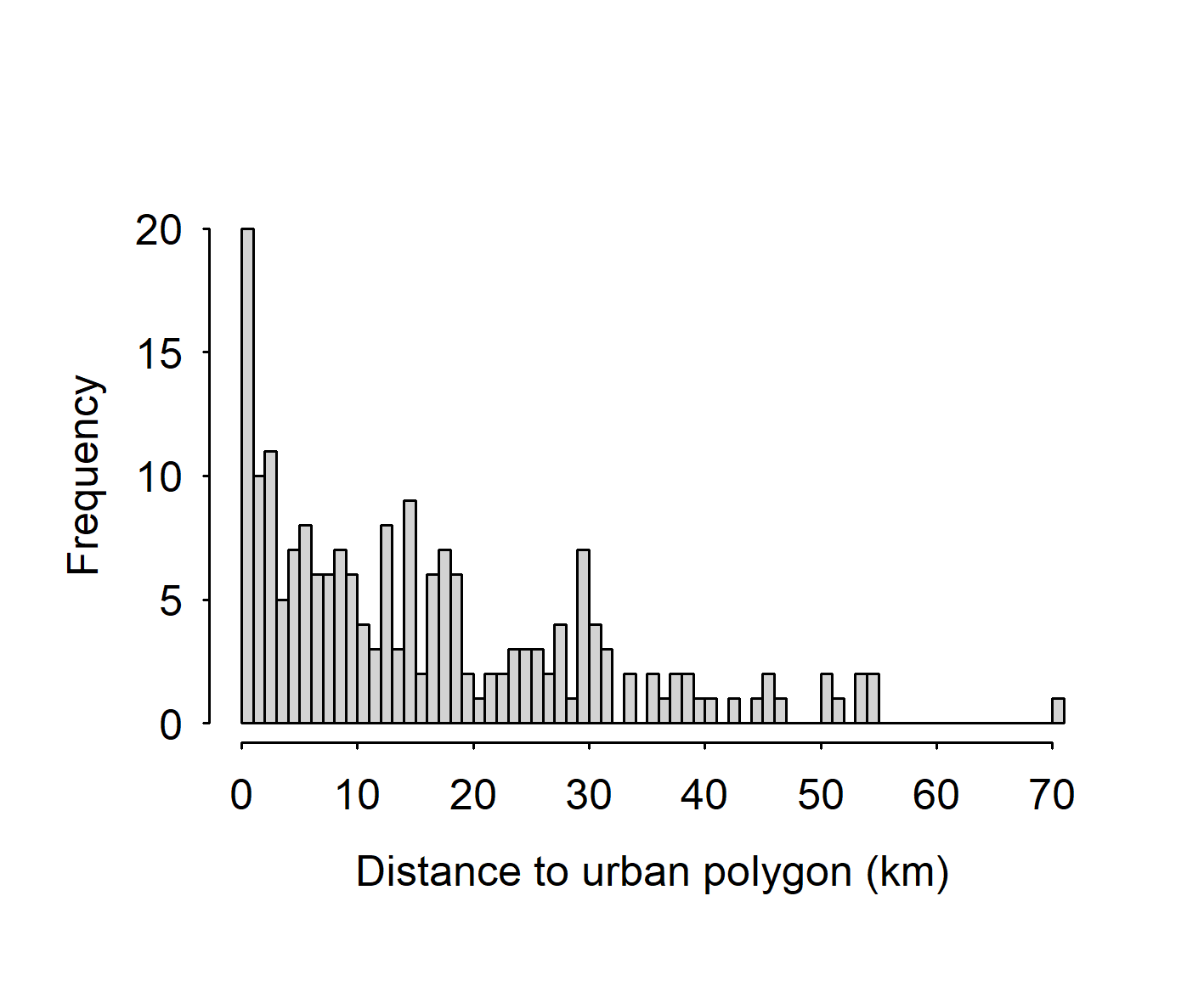

Supplement: S1 Fig — (TIF) [file pone.0259395.s001.tif]

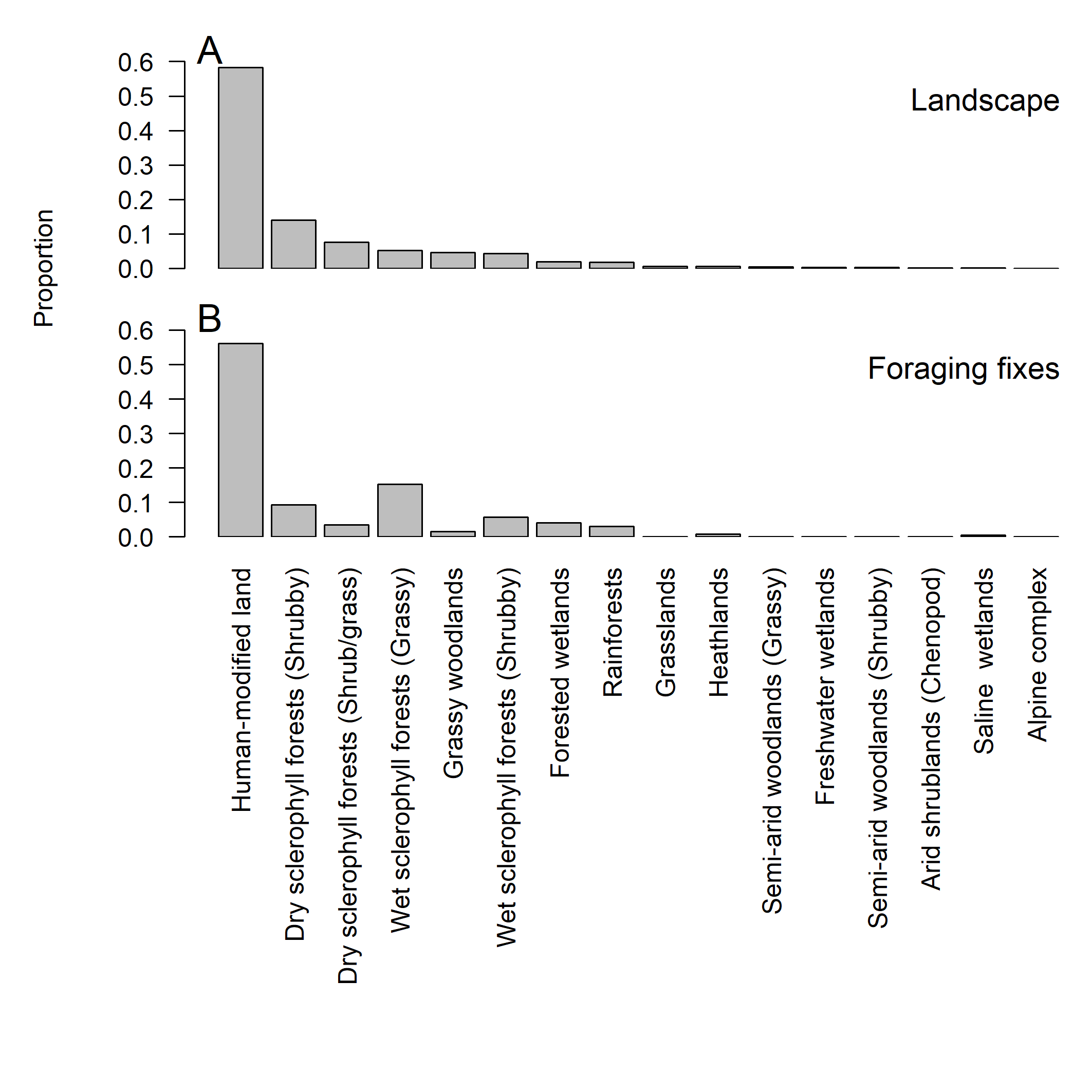

Supplement: S2 Fig — The proportion of (A) each vegetation class in the study area, and (B) of Pteropus poliocephalus foraging positional fixes (n = 4,233) recorded in each vegetation class. Satellite tracking data was collected between 2012–2017 and is representative of 98 individuals. (TIF) [file pone.0259395.s002.tif]

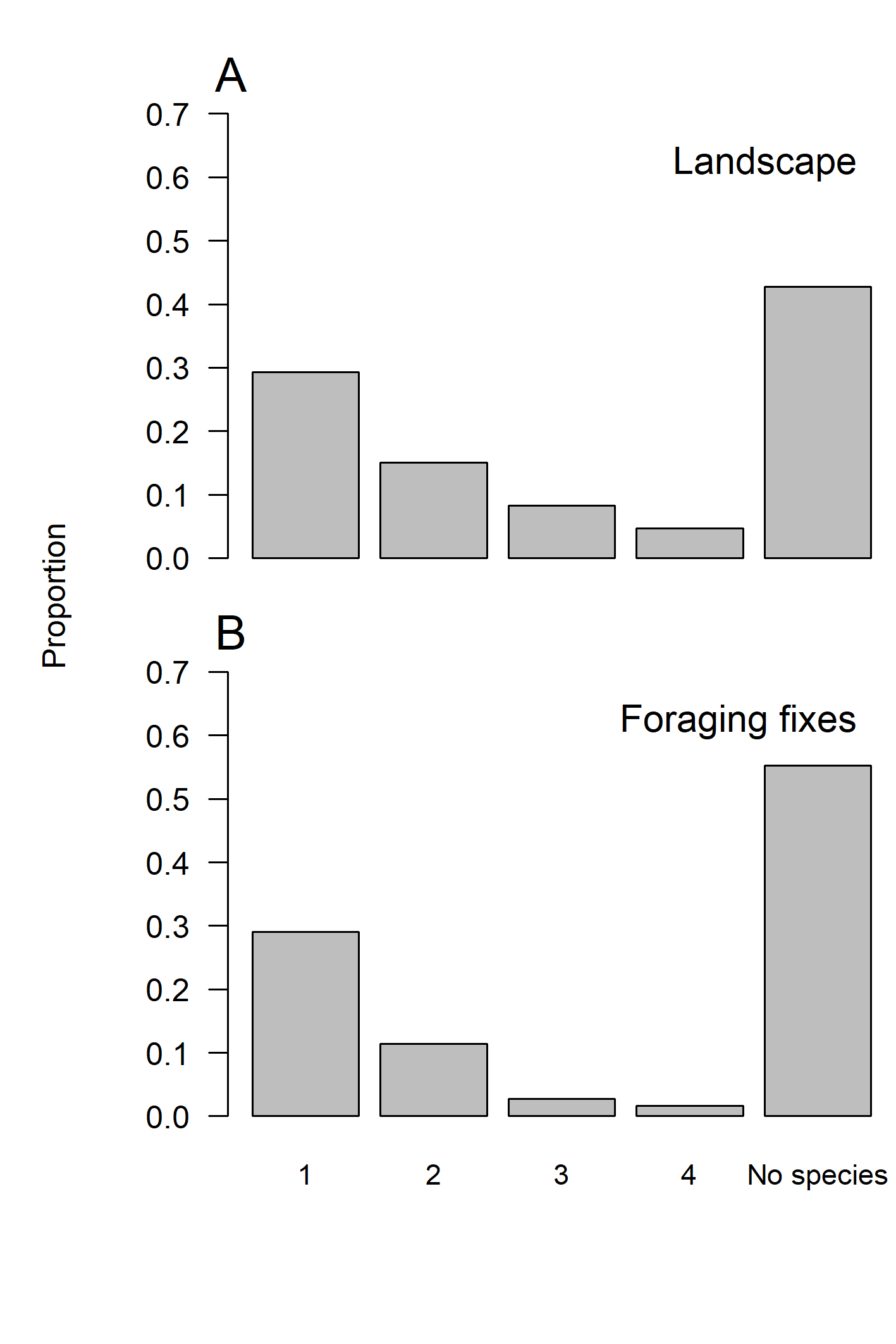

Supplement: S3 Fig — The proportion of (A) each habitat quality rank in the area sampled, and (B) of foraging positional fixes (n = 3,773) recorded in each habitat quality rank. Habitat quality was ranked from 1–4: where 1 is good quality foraging habitat, rank 4 is poor quality foraging habitat, and ‘No species’ is habitat where the recorded dominant and subdominant plant species are not part of the known P. poliocephalus diet. (TIF) [file pone.0259395.s003.tif]

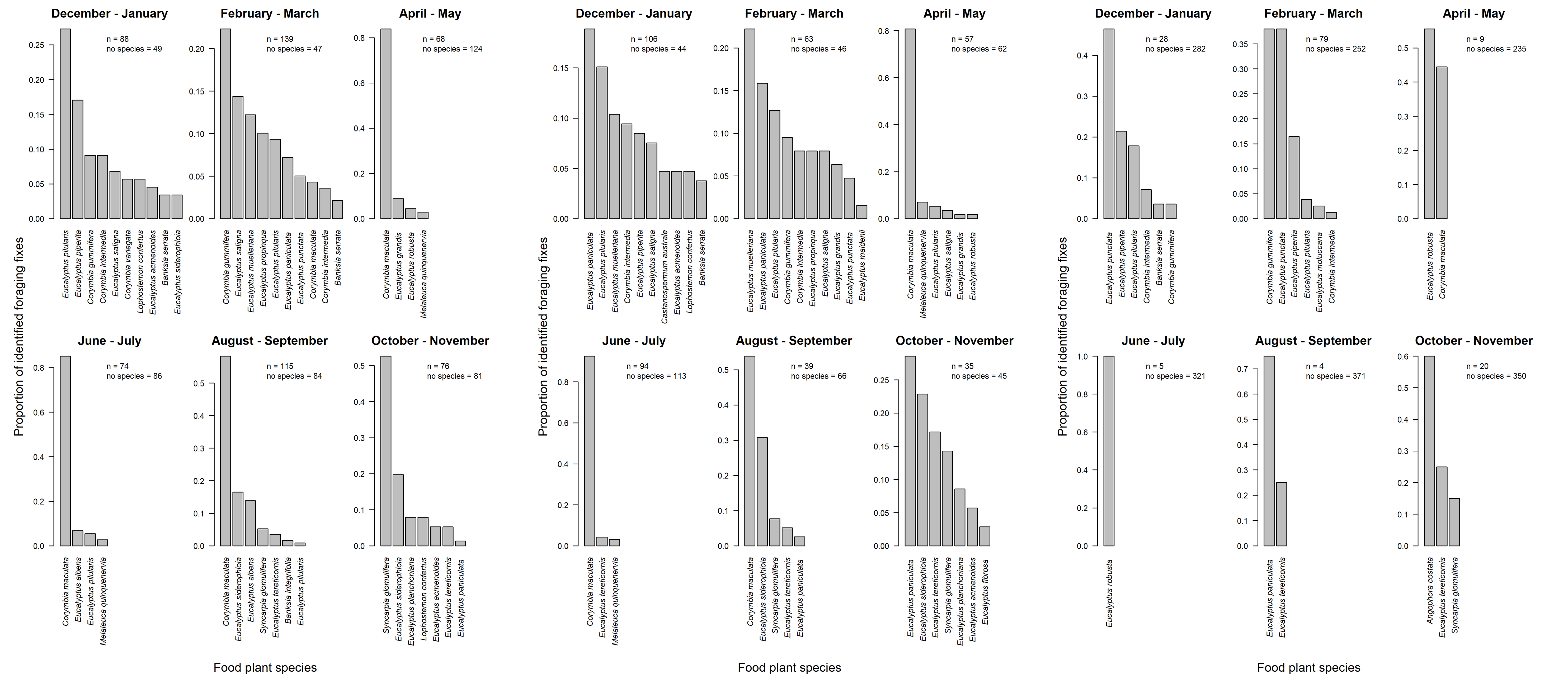

Supplement: S4 Fig — A. Likely P. poliocephalus food plant species in each bi-month where individuals roosted in non-urban areas. A maximum of 10 different food plant species were included in each graph. n = the number of foraging fixes used to calculate proportions. ‘No species’ indicate additional fixes for the particular bi-month that fell in the sampled area, but for which the recorded dominant and subdominant plant species were not part of the known P. poliocephalus diet. B. Likely P. poliocephalus food plant species in each bi-month where individuals roosted in minor-urban areas. A maximum of 10 different food plant species were included in each graph. n = the number of foraging fixes used to calculate proportions. ‘No species’ indicate additional fixes for the particular bi-month that fell in the sampled area, but for which the recorded dominant and subdominant plant species were not part of the known P. poliocephalus diet. C. Likely P. poliocephalus food plant species in each bi-month where individuals roosted in major-urban areas. A maximum of 10 different food plant species were included in each graph. n = the number of foraging fixes used to calculate proportions. ‘No species’ indicate additional fixes for the particular bi-month that fell in the sampled area, but for which the recorded dominant and subdominant plant species were not part of the known P. poliocephalus diet. (TIF) [file pone.0259395.s004.tif]

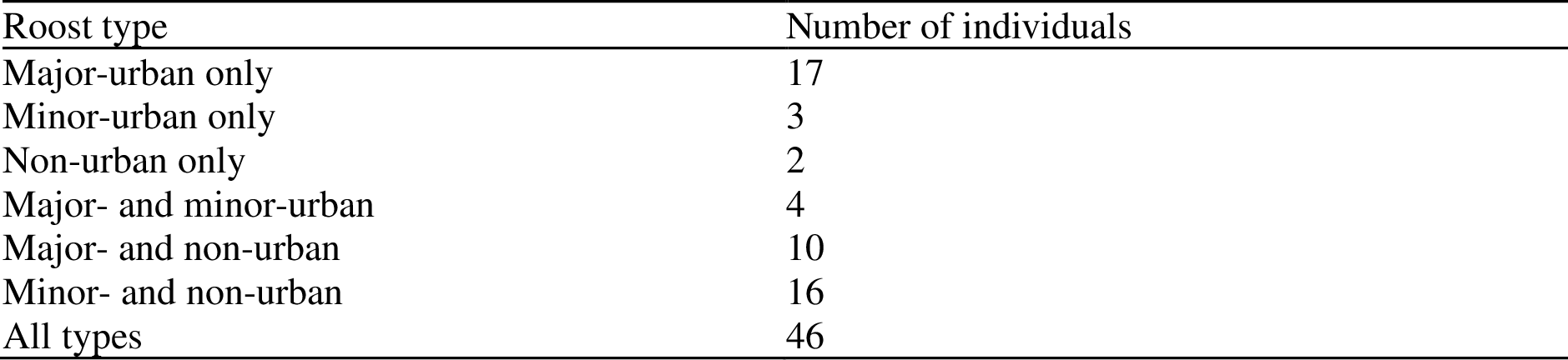

Supplement: S1 Table — (TIF) [file pone.0259395.s005.tif]

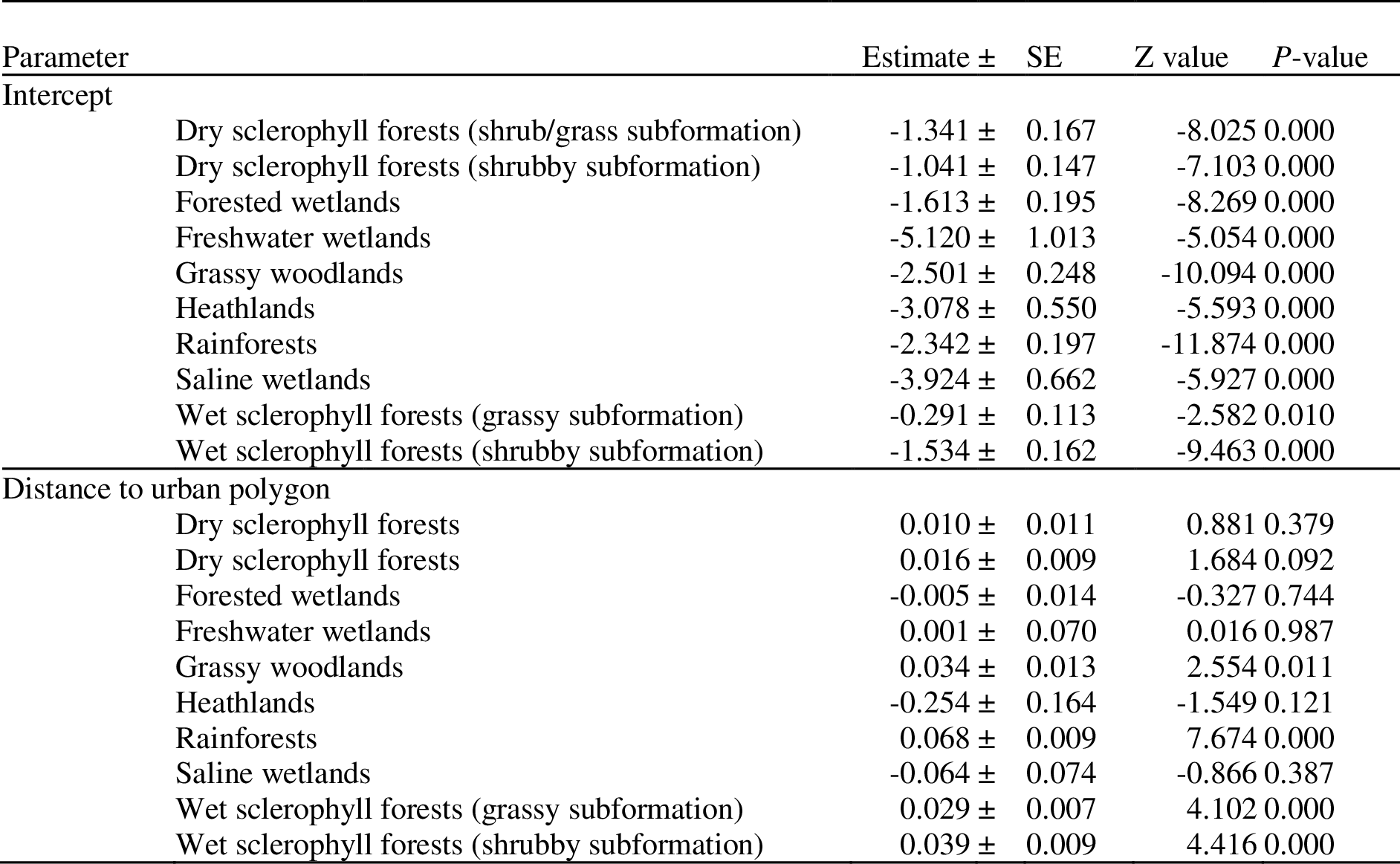

Supplement: S2 Table — Cleared land is the reference category. (TIF) [file pone.0259395.s006.tif]
